# The role of microglial autophagy in Parkinson’s disease

**DOI:** 10.3389/fnagi.2022.1039780

**Published:** 2022-11-01

**Authors:** Rui Zhu, Yuyi Luo, Shangang Li, Zhengbo Wang

**Affiliations:** ^1^State Key Laboratory of Primate Biomedical Research, Institute of Primate Translational Medicine, Kunming University of Science and Technology, Kunming, China; ^2^Yunnan Key Laboratory of Primate Biomedical Research, Kunming, Yunnan, China

**Keywords:** Parkinson’s disease, microglia, microglial autophagy, microglial phagocytosis, neuroinflammation

## Abstract

Parkinson’s disease (PD) is the second most common neurodegenerative disease. Studies have shown that abnormal accumulation of α-synuclein (α-Syn) in the substantia nigra is a specific pathological characteristic of PD. Abnormal accumulation of α-Syn in PD induces the activation of microglia. Microglia, which are immune cells in the central nervous system, are involved in the function and regulation of inflammation in PD by autophagy. The role of microglial autophagy in the pathophysiology of PD has become a hot-pot issue. This review outlines the pathways of microglial autophagy, and explores the key factor of microglial autophagy in the mechanism of PD and the possibility of microglial autophagy as a potential therapeutic target for PD.

## Introduction

Parkinson’s disease (PD) is the second most prevalent neurodegenerative disease and was first reported by James Parkinson in 1817. PD is characterized principally by resting tremor, motor bradykinesia, rigidity, and postural instability, as well as a variety of additional motor and nonmotor symptoms (Jankovic, 2008; Armstrong and Okun, 2020). In 2016, an estimated 6.1 million people were diagnosed with PD worldwide, which is 2.4 times higher than in 1990 (GBD 2016 Parkinson's Disease Collaborators, 2018), as the world population ages and lives longer. Currently, the scientific community is becoming increasingly interested in PD.

In a healthy organism, the homeostasis of the central nervous system (CNS) is dependent on the interactions of various nerve cells. However, in the CNS of PD patients, there is an aberrant build-up of α-synuclein (α-Syn) and a cascade effect of gradual neuronal damage that breaks the appropriate balance, which leads to inflammation in the CNS (Hirsch, 1992). Autophagy is an evolutionarily conserved degradation pathway that is responsible for the digestion and recycling of the majority of intracytoplasmic proteins and organelles. Autophagy maintains homeostasis by delivering cytoplasmic materials to the lysosome for degradation (Bonam et al., 2021). Due to poor autophagy, inappropriately aggregated α-Syn in the CNS of PD patients cannot be removed and accumulated (Aflaki et al., 2017; Karabiyik et al., 2017). Overall, dysregulation of autophagy is thought to play an important role in the abnormal aggregation of α-Syn and the exacerbation of Parkinson’s disease.

Microglia were discovered more than a century ago by using silver carbonate staining (Andres-Barquin, 2002). Microglia are CNS-specific immune cells that play an immunological role in the CNS comparable to that of macrophages, interact with neurons, and conduct a variety of tasks in the CNS (Leng and Edison, 2021). Recent research shows that microglial autophagy is involved in the function and regulation of inflammation in the CNS (Su et al., 2016; Bussi et al., 2017). These findings implied that dysregulation of autophagy in microglia may impact innate immune activities, including phagocytosis and inflammation, which, in turn, contribute to illnesses associated with neuroinflammation (Derecki et al., 2014; Michell-Robinson et al., 2015). To date, many researchers have considered PD to be a neuroinflammatory disease, and the role of microglial autophagy in the pathophysiology of PD has been a hot issue in the field (Cheng et al., 2020; Choi et al., 2020). In this review, we present and highlight the contribution of microglial autophagy to the pathological mechanism of PD and aimed to determine whether microglial autophagy could be a potential target for therapeutic intervention.

## Autophagy in microglia

**Figure 1:**
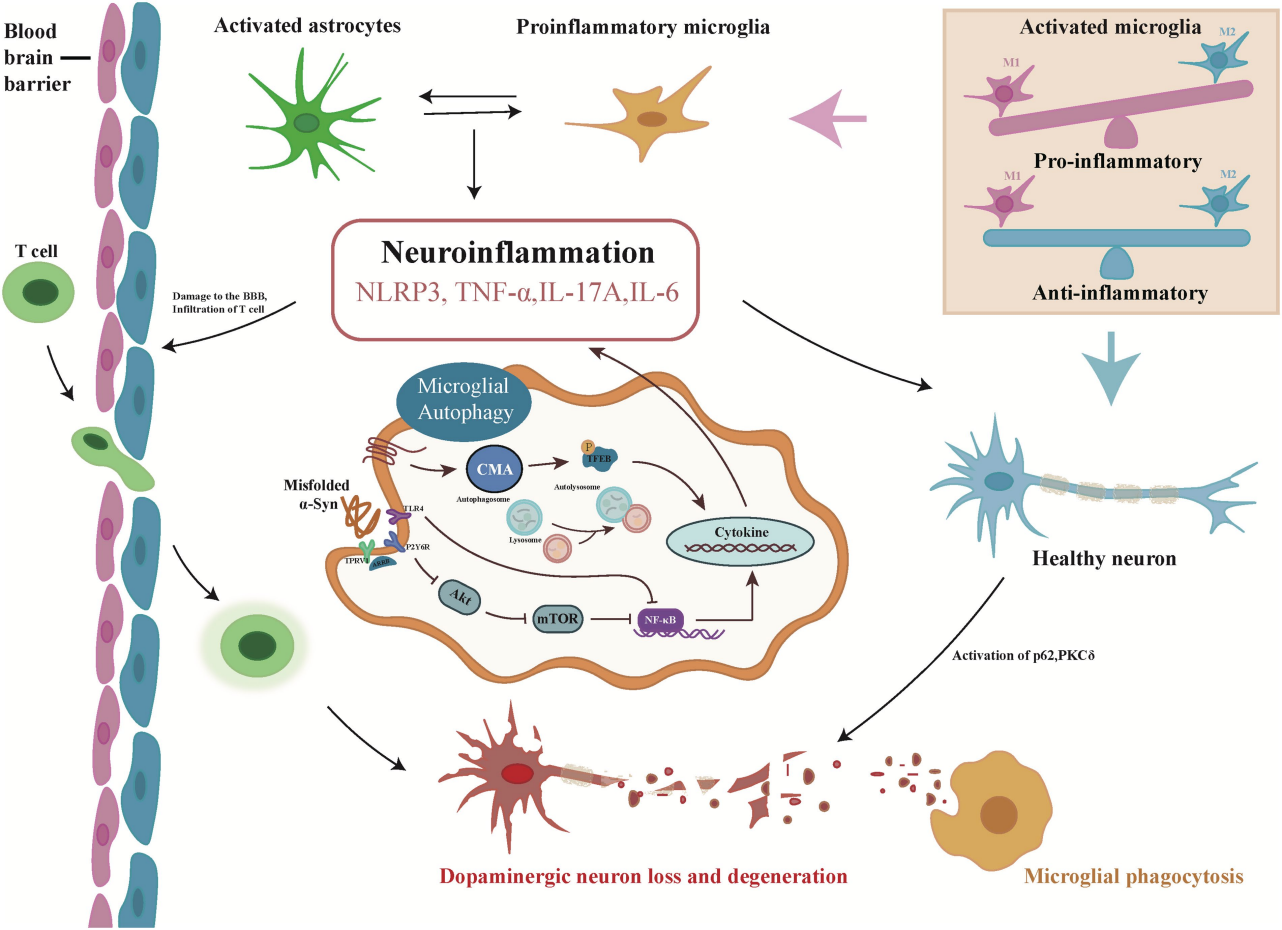
Overview of the microglial autophagy process in Parkinson’s disease. Misfolded α-Syn triggers autophagy in microglia, induces neuroinflammation and releases pro-inflammatory cytokines through canonical (Akt/mTOR), noncanonical (TLR4), and chaperone-mediated (CMA) autophagy pathways. Inflammatory factors accelerate the damage and loss of dopaminergic neurons by disrupting the blood–brain barrier and activating PKCδ, p62, etc., triggering the next autophagy. In the process, pro-inflammatory cytokines, in turn, activate resting microglia, which further activate astrocytes and accelerate neuroinflammation.

Current research suggests that microglia play a crucial role in neurodegenerative disorders. Microglia, like macrophages, can execute their functions under both normal and pathological conditions, and they can also exert adaptive responses under disease conditions (Liu et al., 2022). Microglia can maintain the normal function of nerve cells through synaptic pruning (Paolicelli et al., 2011), and aberrations in the function of microglia during the phagocytosis of synapses may contribute to synaptic loss and neurodegeneration. In addition, microglia play an important role in clearing away apoptotic or necrotic cells and destroying aberrant protein clumps, such as β-amyloid (Aβ) and aggregated α-Syn (Spangenberg et al., 2016; Yuan et al., 2022). In general, microglia use phagocytosis to participate in autophagy in the CNS, which eliminates the effects of the inflammatory response in the organism and maintains homeostasis (Figure 1). Microglial autophagy is the function of carrying protein aggregates and damaged organelles into lysosomes, which includes autophagosome formation and degradation. Autophagosome biogenesis is initiated upon either autophagy-inducing signals or autophagy receptors binding to their targets. Membrane nucleation occurs at specific locations in phagocytes, which then expand and close to autophagosomes (Hu and Reggiori, 2022). The biogenesis of autophagosomes depends on the function of autophagy-related (ATG) proteins (Nakatogawa, 2020). The autophagosome transfers the encapsulated material into the lysosome through fusion of its outer membrane with the lysosomal limiting membrane, forming an autolysosome. Autolysosomes will degrade autophagic cargo as well as the inner autophagosome membrane (Lőrincz and Juhász, 2020). Autophagic flux is primarily related to the intensity of autophagy being induced and the rate of autophagosome fusion digestion by lysosomes, and it is now believed that dysregulated autophagic flux appears to be a major factor in the neuropathology of PD. It was found that in dopaminergic neurons with α-Syn misfolded, lysosomes appear dysfunctional and the rate of autophagosome clearance is decreased (Chu et al., 2009; Dehay et al., 2010). Activation of transcription factor EB (TFEB) drives lysosomal function to clear α-Syn oligomers thereby exerting neuroprotective effects (Settembre et al., 2011; Decressac et al., 2013).

### Microglial phagocytosis and autophagy

Microglia, which are resident brain macrophages, are commonly believed to orchestrate the brain inflammatory response during infections and disease (Sierra et al., 2013). The most significant functional similarity between microglia and macrophages is their ability to perform phagocytosis, which involves three primary steps: recognition, endocytosis, and digestion (Wolf et al., 2017). Phagocytosis is initiated by the activation of membrane receptors, which directly recognize the target to be phagocytosed. In neurological diseases, it has been shown that autophagy can be affected by interfering with the phagocytic function of microglia. Studies have shown that autophagy can regulate phagocytosis through various steps in the phagocytic cascade (Li G. et al., 2021). Restored microglial phagocytosis after peripheral interferon-beta (IFN-β) injection can consequently increase the initiation of brain autophagy (He et al., 2020). Some neurological medications, such as the antidepressant fluoxetine, can increase autophagy further by boosting microglia phagocytosis, thereby targeting neuroinflammation and exerting neuroprotective effects (Park et al., 2021). Berglund discovered that autophagy-related phagocytosis of microglia was critical for recovery from neuroinflammation. Microglia dominate the destruction and removal of myelin sheaths through atypical forms of autophagy in this way and are involved in recovery from demyelinating diseases (Berglund et al., 2020).

### Mechanism of microglial autophagy

Microglia are CNS-specific phagocytes that can perform phagocytosis by rapidly extending and retracting protrusions to recognize aberrant cells or chemicals in the environment (Davalos et al., 2005). Microglia perform autophagy in both canonical and noncanonical manners (Jülg et al., 2020).

Canonical autophagy is the sequential assembly of the autophagic machinery *via* recognition-phagocytosis-digestion. Microglia phagocytose redundant cellular components and deliver them to lysosomes to complete autophagy (Hickey and Kimura, 1988; Lee et al., 2019), and the Unc-51-like autophagy activating kinase complex, class III phosphatidylinositol 3-kinase (PI3KC3) complex, and ATG9/PI3P effector protein recruitment are involved. Song used lipopolysaccharide (LPS) to induce the overexpression of miR-Let7A in microglia and discovered that miR-Let7A may have a role in microglial autophagy during CNS inflammation (Song et al., 2015). Fabbrizio discovered that P2X7 increased the expression of the autophagy marker LC3II *via* the mTOR pathway in Amyotrophic lateral sclerosis rats, thereby modulating autophagy in microglia (Fabbrizio et al., 2017). Wang increased Ccl5 and Cxcl10 expression in a non-p53-dependent manner, indicating that microglial autophagy plays an important role in controlling neurogenesis and restricting local immune responses in postnatal neural stem cells in a noncell-autonomous manner (Wang et al., 2017). Jin discovered that tumor necrosis factor-α (TNF-α) inhibited microglial autophagy *via* the AKT/mTOR signaling pathway; however, enhanced autophagy could promote microglial polarization to the M2 phenotype and promote the resolution of inflammation in PD (Jin et al., 2018). Li showed that a miR-223 inhibitor induced autophagosome and autolysosome production in microglial BV2 cells *via* the ATG16L1-LC3 pathway in response to LPS stimulation (Li et al., 2019). Sakai found that defective autophagy in microglia of the prefrontal cortex exacerbated repeated social defeat (RSD)-induced social avoidance (Sakai et al., 2022).

Unlike canonical autophagy, noncanonical pathways do not require the hierarchical contribution of all ATG proteins. A number of noncanonical autophagic pathways have been identified in microglia (Lee et al., 2019). For instance, Choi discovered a neuroprotective function of microglia in the clearance of α-Syn *via* Toll-like receptors 4 (TLR4)-nuclear factor kappa-B (NF-κB)-p62-mediated synucleinphagy (Choi et al., 2020). Wang identified a role for microglial NF-κB signaling in regulating tau protein diffusion and tau lesion toxicity (Wang et al., 2022). The above studies show that in microglia, both canonical and noncanonical autophagy pathways can be regulated by different methods.

### Microglial autophagy and neuroinflammation development

Phagocytic microglia promote autophagy in the CNS mostly due to neuroinflammation. Studies have shown that microglia use protrusions to communicate with neurons and other glial cells during movement according to a variety of internal environments (Davalos et al., 2005; Ladeby et al., 2005; Fourgeaud et al., 2016). Under physiological conditions, this motor communication ability of microglia enables them to act as scavengers, maintain the balance of the internal environment, remove metabolites, and regulate neurogenesis (Colonna and Butovsky, 2017; Stratoulias et al., 2019). Under endogenous or exogenous pathological conditions, microglia, similar to macrophages, will change from a quiescent state to an “activated” state, undergo morphological changes to exert phagocytic function, induce an inflammatory response, and release a variety of pro-inflammatory cytokines. Therefore, activated microglia determine the fate of other surrounding nerve cells to a certain extent, maintain the healthy state of the tissue environment and overcome the development of pathologies such as neuroinflammation (Miyamoto et al., 2016; Hagemeyer et al., 2017; Kam et al., 2020; Tu et al., 2021). Activated microglia can often be categorized as M1 or M2 cells. The activation of M1 microglia is called classical activation. M1 microglia are cytotoxic to neurons and other glial cells by producing a large number of cellular mediators, such as proteases, pro-inflammatory cytokines, and reactive oxygen species (ROS), which lead to pro-inflammatory responses (Daher et al., 2014). Pro-inflammatory cytokines could activate M1 microglia to express pro-inflammatory factors, such as IL-1β, TNF-α, IL-6, nitric oxide (NO) and protease have adverse effects on neurodegenerative diseases (Glass et al., 2010; Tang and Le, 2016). It is now believed that the degree of M1 microglia activation correlates with the loss of dopaminergic neurons in early PD (Ouchi et al., 2005). TNF-α, NO and IL-1β originating from M1 microglia can regulate the neuroinflammatory process in PD (Rojanathammanee et al., 2011). M2 microglia are considered to be another activated state that are capable of participating in the phagocytosis of cellular debris or damaged neurons and release various neurotrophic factors and cytokines, thereby exerting anti-inflammatory effects (Cherry et al., 2014). IL-4, IL-10, IL-13, and transforming growth factor-β could activate M2 microglia to release various cytokines, such as insulin-like growth factor 1 and frizzled receptor 1 (Glass et al., 2010; Tang and Le, 2016), and these cytokines may be involved in neuroprotection and tissue healing. M1 and M2 microglia can transform into each other under certain circumstances, inflammatory mediators released by M1 microglia are involved in mechanisms of neurodegenerative diseases, and M2 microglia are involved in tissue maintenance and repair. Microglia-mediated neuroinflammation is therefore regarded as a double-edged sword in neurodegenerative diseases, with deleterious and beneficial effects on neurons and the surrounding environment (Guo et al., 2022). Aggregated α-Syn can directly activate microglia toward the M1 phenotype (Tang and Le, 2016). Under hypoxic and inflammatory conditions, inhibition of the mTOR pathway was found to prevent microglial activation, reduce neuroinflammation, and protect neurons (Srivastava et al., 2016).

## Microglial autophagy in PD patients

The cellular and molecular alterations observed in the brains of PD patients have been further studied in PD animal models. Here, we describe microglial changes in PD patients, the mechanism of microglial autophagy associated with α-Syn disaggregation dopaminergic neuron degeneration in animal models of PD, and microglial autophagy as a potential therapeutic target that may help slow the progression of PD.

In 1988, McGeer detected a large number of HLA-DR-positive reactive microglia (macrophages) in the substantia nigra of PD patients, along with LBs (McGeer et al., 1988). Later, Imamura confirmed that M2 microglia were associated with the CNS under aging and pathological conditions; that is, M2 microglia are sensitive indicators of neuropathological changes and are closely associated with damaged neurons and neurites (Imamura et al., 2003; Table 1). To further understand the differences in the transcriptional levels of microglia in PD, Mastroeni used single-cell laser technology to examine microglia in the brains of PD patients and found that microglia in different brain regions have different transcriptional profiles and functionally regulated genes (Mastroeni et al., 2018). However, in general, the RNA-seq data obtained from the brain tissue of PD patients are relatively small, and it is not possible to draw clear conclusions about the correlation between microglial transcriptional heterogeneity and disease. However, these data strongly demonstrate that microglia play an important role in PD.

**Table 1:** Summary of microglial autophagy in PD.

	Pathology	Mechanism	Pharmacological
PD patients	HLA-DR-positive reactive microglia (macrophages) in the substantia nigra, along with LBs (McGeer et al., 1988). M2 microglia appeared near damaged neurons and neurites (Imamura et al., 2003).	M2 microglia associated with the CNS in aging and pathological conditions (Imamura et al., 2003).	——
PD animal models	Activation of microglia occurs in brain regions where α-Syn aggregates (Gerhard et al., 2006). Microglia are the target cells of exogenous exosomes (Xia et al., 2019). Excessive activation of microglia can exacerbate neuroinflammation (Guan et al., 2021). Microglia have the highest density in the substantia nigra (Yang et al., 2013). Activation of NF-κB in microglia is critical for regulating pro-inflammatory cytokine levels in the substantia nigra (Ghosh et al., 2007).	Microglia clear misfolded α-Syn: TLR4-NF-κB-p62 pathway (Choi et al., 2020). Pro-inflammatory: PKCδ (Gordon et al., 2016), PGC-1α (Guan et al., 2021), ARRB1 (Fang et al., 2021), and NLRP3 (Chen et al., 2021). Anti-inflammatory: DJ-1 (71), Th17 (Liu et al., 2019), ARRB2 (Fang et al., 2021).	Accelerate the degradation of α-Syn: TRPV1 (Yuan et al., 2022). Reduced pro-inflammatory response: Blocking P2Y6R (Yang et al., 2017), attenuating NLRP3 inflammasome (Ahmed et al., 2021). Anti-inflammatory drugs that exert neuroprotective effects: NLY01 (Yun et al., 2018), nilotinib (Wu et al., 2021b), and cannabidiol (Wu et al., 2021a).

## Mechanism of microglial autophagy in PD animal models

α-Syn aggregates in substantia nigra dopamine (DA) neurons are important in the pathogenesis of PD (Ghosh et al., 2017). Recent studies have shown that activated microglia accelerate pathological α-Syn accumulation in DA neurons (George et al., 2019; Tan et al., 2020). Microglia exposed to abnormal aggregation of α-Syn can form F-actin-dependent intercellular junctions, thereby establishing cellular networks that can transfer abnormally aggregated α-Syn from overloaded microglia to adjacent inactivated microglia for their rapid and efficient degradation. This degradation process could reduce the inflammatory response of microglia and increase their survival (Scheiblich et al., 2021). The mechanism by which microglia improve the clearance of pathogenic α-Syn aggregates by creating an “on-demand” functional network has been elucidated (Table 1). A study showed that microglia can transmit α-Syn through exosomes and aggravate the accumulation of α-Syn, indicating that the secretion of exosomes can alter the progression of PD (Xia et al., 2019). Guo found that α-Syn oligomers were present in microglia-derived exosomes in the cerebrospinal fluid of patients with Parkinson’s disease, which could induce α-Syn aggregation in neurons, indicating that microglia can accelerate α-Syn aggregation in Parkinson’s disease *via* exosomes (Guo et al., 2020). Misfolded α-Syn in PD can alter TLRs expression in microglia and modulate persistently activated protein kinase Cδ (PKCδ) dependent nuclear displacement of NF-κB (Li Y. et al., 2021). Choi identified that microglia mediate synaptic phagocytosis through the TLR4-NF-κB-p62 pathway, thereby clearing α-Syn for neuroprotective effects (Choi et al., 2020; Figure 1).

Neuroinflammation and oxidative stress play important roles in the pathogenesis of PD. During these processes, microglia are activated and involved in the progressive degeneration and loss of nigrostriatal DA neurons (Table 1). PKCδ drives a persistent inflammatory response in microglia, leading to dopaminergic neuronal neurotoxicity, which, in turn, causes neurobehavioral deficits (Gordon et al., 2016). Mutations in the oxidative stress sensor DJ-1 cause family PD. Knockdown of DJ-1 in microglia increased neurotoxicity in DA neurons and significantly increased α-Syn-induced secretion of pro-inflammatory cytokines (Nash et al., 2017). The study showed that in the microglia of PD mice, the expression level of PGC-1α (peroxisome proliferator-activated receptor-γ coactivator-1α), which is closely related to the inflammatory response, was increased, and PGC-1α/Iba1 colocalization was increased. The downregulation of PGC-1α expression can inhibit the activity of microglia and reduce the activation of microglia, thereby protecting DA neurons (Guan et al., 2021). Further study showed that when the blood–brain barrier was disrupted, effector T cells transferred into the brain and aggravated the degeneration of nigrostriatal DA neurons, the activation of microglia and motor impairment (Liu et al., 2017), and inhibiting IL-17A secretion by Th17 cells in the substantia nigra ameliorated the above symptoms. IL-17A exacerbates DA neuron loss only in the presence of microglia, suggesting a crucial role for microglia in DA neuron degeneration (Liu et al., 2019). Subbarayan examined T-cell-deficient and T-cell-normal rats and showed that T cells may be directly involved in α-Syn-mediated loss of DA neurons in PD and activation of microglia (Subbarayan et al., 2020). The expression of β-repressor protein 1 (ARRB1) and β-repressor protein 2 (ARRB2) in microglia is reciprocally regulated, and Fang found that ARRB1 ablation ameliorated the pathological features of PD, whereas ARRB2 knockout aggravated the pathological features of PD (Fang et al., 2021). Nucleotide-binding oligomerization domain-, leucine-rich repeat-, and pyrin domain-containing 3 (NLRP3), the most well-studied inflammasome, is abundantly expressed in the activated microglia and has gained considerable attention (Wu A. G. et al., 2021). The excessive activation of NLRP3 inflammasome could impair microglial autophagy and further aggravates the pathogenesis of neurodegenerative diseases. α-Syn aggregates activate NLRP3 inflammatory bodies in microglia through interaction with TLRs and activation of NF-κB (Béraud et al., 2011; Panicker et al., 2019). Chen investigated whether the p38-TFEB pathway promoted microglial activation by inhibiting CMA-mediated NLRP3 degradation in Parkinson’s disease, which could be a potential therapeutic strategy for PD (Chen et al., 2021; Figure 1).

## Autophagy of microglia and PD therapeutic advances and future directions

Numerous studies have been conducted on the role of microglia-induced neuroinflammation and microglial autophagy in the pathogenesis of PD to identify new strategies for the treatment of PD through pathways related to microglial activation and neuroinflammation. It was found that the stimulation of P2Y6R accelerated the activation of microglial cells and the release of pro-inflammatory cytokines; that is, blocking P2Y6R may inhibit microglial activation and phagocytosis (Yang et al., 2017). Activation of NLRP3-mediated inflammatory responses impairs microglial autophagy and exacerbates 1-methyl-4-phenyl-1,2,3,6-tetrahydropyridine (MPTP)-induced pro-inflammatory responses to PD-like symptoms (Cheng et al., 2020; Qin et al., 2021). Ahmed uses andrographolide to enhance microglial activation by attenuating NLRP3 inflammasome activation in microglia, enabling the rescue of dopaminergic neurons and ameliorating behavioral deficits in animals (Ahmed et al., 2021). Yuan used photothermal nanomaterials to control the opening of transient receptor potential cation channel subfamily V member 1 (TRPV1) channels on the surface of microglia to enhance phagocytosis by microglia and accelerate the phagocytosis and degradation of α-Syn, thereby ameliorating PD (Yuan et al., 2022; Figure 1).

Based on related research, the efficacy of some drugs was further explored. NLY01 is a glucagon-like peptide-1 receptor (GLP-1R) inhibitor that can exert neuroprotective effects by directly regulating the activated microglia-mediated transformation of astrocytes to the A1 neurotoxic phenotype in PD (Yun et al., 2018). Nilotinib was found to inhibit microglial activation and the production of pro-inflammatory cytokines and mediators by inhibiting the NF-κB signaling pathway. The anti-inflammatory properties of nilotinib may further reduce inflammatory neurotoxicity to protect against DA neuron loss, indicating its potential use in PD (Wu et al., 2021b). Cannabidiol (CBD) can inhibit IL-6 release and improve microglial activation, and there is a correlation between CBD-induced signal transducer and activator of transcription 3 (STAT3) and NF-κB phosphorylation, indicating that CBD may have therapeutic potential for treating neurological diseases involving neuroinflammation (Wu et al., 2021a; Table 1).

## Conclusion

In this review, we described the role of microglial autophagy in PD pathogenesis and explored potential therapeutic strategies for PD. Microglia participate in the process of autophagy through phagocytosis and regulate the occurrence and development of neuroinflammation (Tang and Le, 2016; Table 1). In PD, α-Syn and neuroinflammation trigger signaling pathways such as NLRP3, which activate microglia (Chen et al., 2021). Activated microglia alter the aggregation and propagation of misfolded α-Syn through autophagy to further influence neurons and neurobehavior. In this process, only properly activated microglia contribute to the removal of harmful substances, and excessive autophagy and/or dysregulated autophagic flux can lead to neurotoxicity (Qin et al., 2021). It is well known that autophagy in microglia exerts neuroprotective effect by regulating phagocytosis and inflammation (Tang and Le, 2016; He et al., 2020); however, the mechanism of autophagy in microglia is complex. Therefore, further examination of the mechanism of microglial autophagy in PD can help us identify better strategies regarding microglial autophagy in the field of PD therapy.

## Author contributions

All authors listed have made a substantial, direct, and intellectual contribution to the work and approved it for publication.

## Funding

The authors are grateful to be supported by the National Natural Science Foundation of China (31960120) and the Applied Basic Research Programs of Science and Technology Commission Foundation of Yunnan Province (202105AC160041 and 2018FB052).

## Conflict of interest

The authors declare that the research was conducted in the absence of any commercial or financial relationships that could be construed as a potential conflict of interest.

## Publisher’s note

All claims expressed in this article are solely those of the authors and do not necessarily represent those of their affiliated organizations, or those of the publisher, the editors and the reviewers. Any product that may be evaluated in this article, or claim that may be made by its manufacturer, is not guaranteed or endorsed by the publisher.
